# Incarcerated sigmoid colon cancer in an inguinal hernia sac associated with an abdominal wall abscess: a case report

**DOI:** 10.1186/s40792-019-0742-2

**Published:** 2019-12-05

**Authors:** Hironori Mizuno, Hidemasa Nagai, Shingo Maeda, Hideo Miyake, Yuichiro Yoshioka, Norihiro Yuasa

**Affiliations:** 1 Department of Gastrointestinal Surgery, Japanese Red Cross Nagoya First Hospital, Nagoya, 453-8511 Japan; 2 Department of Surgery, Japanese Red Cross Nagoya First Hospital, Nagoya, 453-8511 Japan

**Keywords:** Colorectal cancer, Inguinal hernia, Abscess

## Abstract

**Background:**

An inguinal hernia is a common disease; however, a malignant tumor within the inguinal hernia sac is rare, and perforated colon cancer in the hernia sac is extremely rare.

**Case presentation:**

A 73-year-old man presented to our hospital with high fever and painful bulging of the lower abdomen. Computed tomography showed air-containing fluid in the abdominal wall, as well as localized wall thickness of the sigmoid colon in the left groin. An emergency operation revealed a huge subcutaneous abscess and a hard mass of the sigmoid colon within an indirect inguinal hernia sac. Sigmoidectomy and hernia repair using the Marcy method were performed. Lymph node dissection was performed through a transrectal abdominal incision. Histopathological examination of the resected specimen revealed moderately differentiated adenocarcinoma invading the serosal layer with lymph node metastasis.

**Conclusions:**

Incarcerated inguinal hernia with perforated colon cancer is rare; an emergent operation should accordingly be performed based on infection control, oncological principles, and secure hernia repair.

## Background

An inguinal hernia is a common disease; however, a malignant tumor within the inguinal hernia sac is rare. An inguinal hernia associated with perforated sigmoid colon cancer is extremely rare, and the treatment of this condition is challenging. The incidence of malignant tumors within the inguinal hernia sac is reportedly 0.4–0.5% among the cases with excision [1, 2]. We present a case of incarcerated sigmoid colon cancer in an inguinal hernia sac associated with an abdominal wall abscess.

## Case Presentation

### History, examination, and radiological findings

**Fig. 1:**
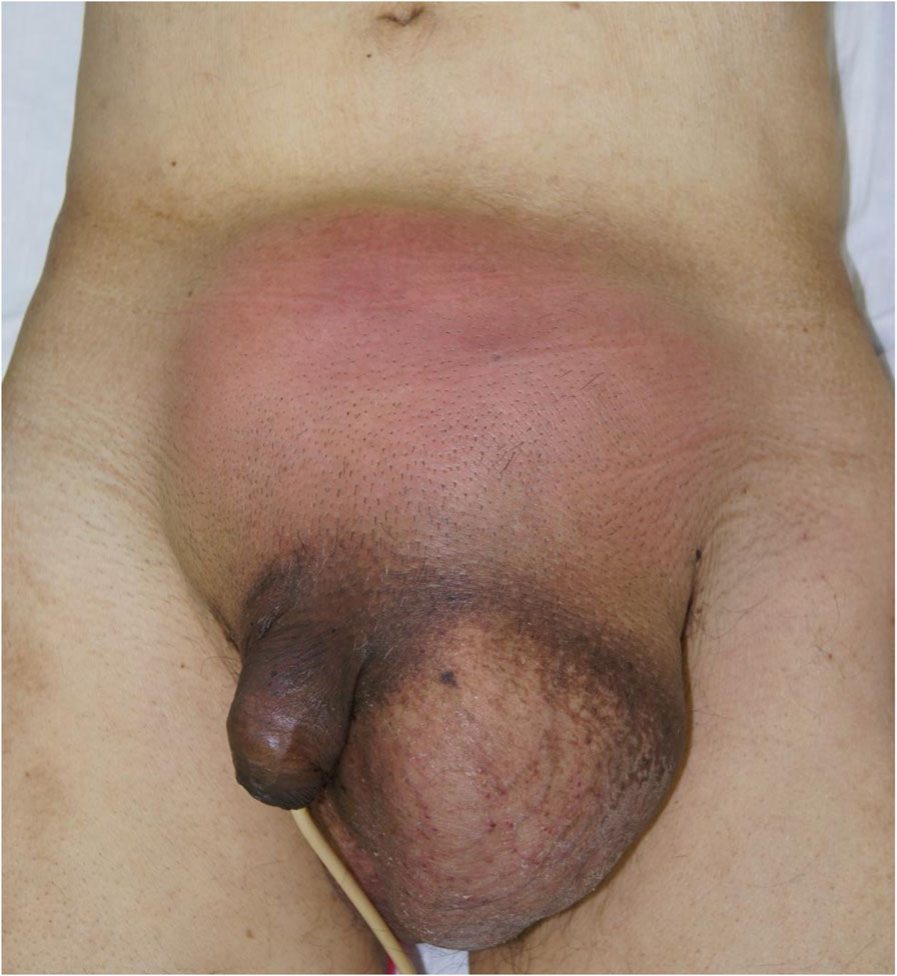
Appearance of the lower abdomen and groin on admission

**Fig. 2:**
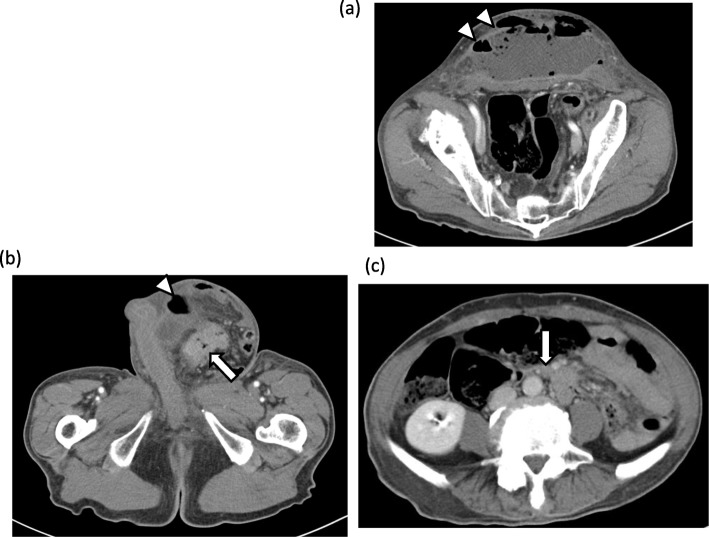
**a**, **b** Computed tomography scan showing air-containing fluid in the lower abdominal wall (arrow head), localized wall thickness of the sigmoid colon in the left groin (arrow), and (**c**) swollen lymph nodes along the IMA and abdominal aorta (arrow)

A 73-year-old man presented to our hospital on May 2017 with painful bulging of the lower abdomen. He was febrile with a temperature of 38.4°C and had a swollen reddish lower abdomen and a large left scrotum (Fig. 1). Laboratory results showed an elevated white blood cell count (38.3 × 10^3^/μL) and C-reactive protein (20.4 mg/dL) and a low hemoglobin level (5.9 mg/dL). Computed tomography (CT) showed air-containing fluid in the abdominal wall (Fig. 2a), localized wall thickness of the sigmoid colon in the left groin (Fig. 2b), and swollen lymph nodes along the inferior mesenteric artery (IMA) and abdominal aorta (Fig. 2c). We suspected a perforated sigmoid colon cancer with abdominal wall abscess due to the incarcerated left inguinal hernia and performed an emergency operation.

### Operation

**Fig. 3:**
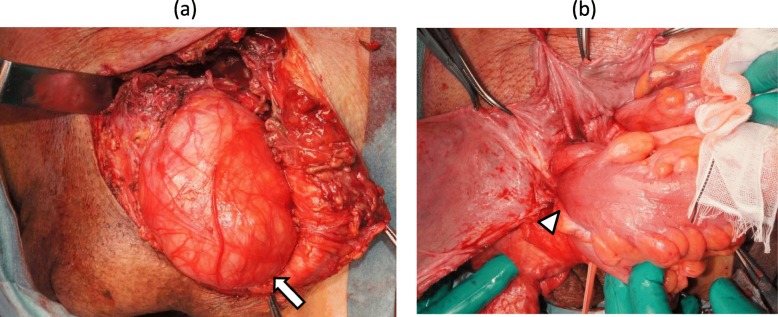
Surgical views showing a large hernia sac (**a**, arrow) and segmental wall thickness of the sigmoid colon within the hernia sac (**b**, arrow head)

**Fig. 4:**
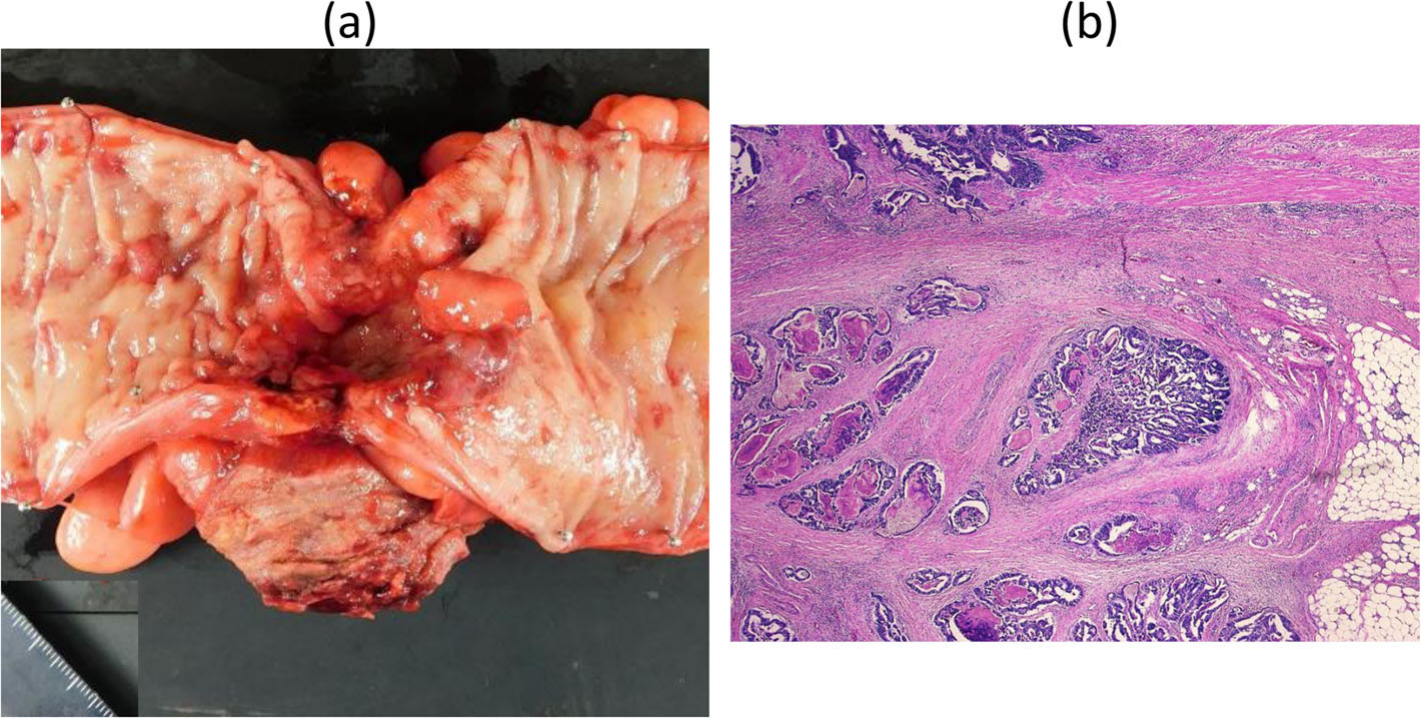
**a** Macroscopic findings of the resected specimen showing a well-demarcated ulcerative protruding lesion. **b** Histological examination showed moderately differentiated adenocarcinoma invading the serosal layer (hematoxylin and eosin staining, x200)

When the left inguinal skin was incised, purulent fluid flowed from a subcutaneous abscess cavity, and a huge hernia sac was identified (Fig. 3a). Upon opening the sac, we observed that a hard mass of the sigmoid colon was tightly adhering to the sac (Fig. 3b). There was no dirty fluid within the hernia sac. There was no sign of circulatory disturbance in the sigmoid colon. The hernial orifice was located on the lateral side of the inferior epigastric artery, indicating an indirect inguinal hernia. Sigmoidectomy with partial resection of the hernia sac adhering to the sigmoid colon was performed, and both the oral and anal colon were placed in the abdominal cavity through the hernial orifice. The inguinal hernia was repaired with the Marcy method (for narrowing the internal inguinal ring) combined with suturing the external oblique aponeurosis to the iliopubic tract (for reinforcing the inguinal canal floor). The abdominal cavity was opened through an additional transrectal abdominal incision, and lymph node dissection in the sigmoid mesentery and colo-colostomy were performed. The lymph nodes along the IMA could not be dissected because of tight tissue, probably due to lymphangitis. Two Penrose drains were placed into the subcutaneous cavity. Macroscopic findings of the resected specimen revealed a well-demarcated, ulcerative protruded lesion (Fig. 4a), which was histologically diagnosed as moderately differentiated adenocarcinoma invading the serosal layer (Fig. 4b) with lymph node metastases. The abdominal wall abscess had developed due to penetration of the sigmoid cancer into the hernia sac.

### Postoperative course

Postoperatively, meropenem 1.5 g/day was administered intravenously, however, the patient developed a large infectious abdominal wound. *Escherichia coli* and *Bacteroides fragilis* were identified by bacterial culture of the abdominal wall abscess. Additional abdominal wall debridement was performed to promote infection control. He was discharged on the 29th postoperative day. At 9 months post-surgery, he had multiple liver metastases and has undergone systemic chemotherapy.

## Discussion

Although inguinal hernia is commonly observed, approximately 10% of this disease is irreducible and can cause bowel obstruction or strangulation [3]. Coexisting malignancy is rarely the cause of irreducibility. Malignancy of the inguinal hernia can be classified as either saccular or intrasaccular [4]. Saccular malignancy is a primary tumor originating from the hernia sac, such as mesothelioma or metastasis to the peritoneum (hernia sac); conversely, the intrasaccular malignancy is a primary tumor of other organs within the sac, such as the one reported here [5]. Moreover, this was the first case of intrasaccular malignancy (0.03%) among the 2893 inguinal hernias that were surgically treated in our department between January 2001 and December 2017.

Our case was specific for developing a huge abscess cavity outside the hernia sac. Benfatto et al. [6] reported a similar case. We speculate the mechanism how the subcutaneous abscess developed as follows: the sigmoid colon carcinoma invaded to peritoneum (hernia sac) and was perforated to the extraperitoneal space (subcutaneous tissue).

We repaired the inguinal hernia with the Marcy method and combined with suturing the external oblique aponeurosis to the iliopubic tract that was because there was no stiff fascia tissue excluding the external oblique aponeurosis because of severe inflammation. If the patient will develop hernia recurrence without infection, hernia repair using a mesh is one of options.

Although primary colonic malignancy within an inguinal hernia sac has rarely been reported, our extensive literature search yielded 12 cases of perforated colonic cancer within an inguinal hernia sac (Table 1) [2, 3, 6–14]. This disease was more common among elderly men and frequently involved the sigmoid colon. The inguinal hernia was predominantly in the left side. Sigmoidectomy with colostomy was performed in most cases. R0 resection was performed in several cases; however, in our case, we failed to execute a potential curative resection because of insufficient lymph node dissection along the IMA. The operations for inguinal hernia were mostly performed without artificial materials. The outcome in the aforementioned cases was limited to short-term results.

**Table 1:** Reported cases of a perforated malignant tumor in an inguinal hernia sac

No	Year	Author	Age	Sex	Hernia Side	Sites of colon cancer	Depth of invasion	Operation	Radicality	Operation for Inguinal hernia	Hospital stay (days)	Outcome (months)	
1	1981	Javor [7]	77	M	Left	S	nd	Sx	nd	nd	-	-	dead
2	1987	Pappas [8]	80	M	Left	S	T4	Sx + colostomy	nd	nd	73	2.4	dead
3	1992	Dewire [9]	77	M	Left	S	T2	Sx + colostomy	R0	nd	-	-	-
4	2003	Kourakalis [3]	79	M	Left	S	T4b	Sx + colostomy	R0	Lichtenstein	10	1	alive
5	2006	Boormans [2]	44	M	Right	S	T3	nd	R0	nd	35	12	alive
6	2006	Benfatto [6]	79	M	Right	C	T4	RHC	R0	Plug	12	18	alive
7	2008	Sakorafas [10]	85	M	Right	S	nd	Sx + colostomy	nd	Bassini	15	-	-
8	2008	Slater [11]	73	M	Left	S	T4	Sx + colostomy	R0	nd	-	-	alive
9	2009	Ruiz-Tovar [12]	67	M	Left	S	T4	Sx	R0	Lichtenstein	7	0.2	alive
10	2010	Ko [13]	84	M	Left	S	T4	Sx + colostomy	nd	nd	5	0.2	dead
11	2013	Tan [14]	63	M	Left	S	T4b	Sx + colostomy	R0	Primary suture	-	0.3	alive
12		Mizuno	73	M	Left	S	T4a	Sx	R2	Marcy	29	25	alive

C, cecum; S, sigmoid colon; Sx, sigmoidectomy; RHC, right-sided hemicolectomy; nd, not described

Clinical presentation of perforated malignant tumors within an inguinal hernia sac mimics strangulation of the incarcerated bowel. This critical condition, with the associated infection and circulatory disturbance, can have a significant effect on the clinical outcome. Surgery should immediately be performed because any delay can lead to fatal complications, such as necrotizing fasciitis and sepsis [9]. Surgeons should plan surgery in advance or during surgery with a clear diagnosis because perforations of malignant tumors and enterocutaneous fistulas are reportedly associated with high morbidity and mortality rates [15]. Various surgical approaches have been reported for this disease; however, the optimal approach has not yet been determined. A standard inguinal hernia repair via a groin incision and colectomy through another laparotomy is one surgical option. Advances in imaging modalities have led to the precise diagnosis of hernia content. Although open surgery was performed in 11 of the reported cases with perforated colon cancer, Pernazza et al. reported the use of laparoscopic surgery for a primary colon carcinoma incarcerated in an inguinal hernia [16]. Irrespective of the surgical approach used, the operation should be performed considering the oncological principles, secure hernia repair, and infection control.

It is not clear whether it is necessary to screen bowel malignancy in patients with an inguinal hernia. Avidan et al. reported no significant association between inguinal hernia and colon cancer [17, 18]. However, bowel examination might be beneficial for male elderly patients presenting with a swollen groin to avoid a challenging performing surgery for a perforated malignant tumor within an inguinal hernia sac.

## Conclusions

In this report, we discussed a rare case of perforated colon cancer in the hernia sac in a 73-year-old man. Although incarcerated inguinal hernia with perforated colon cancer is rare, it should be considered in patients with an inflammatory irreducible groin mass. Precise diagnosis is important, and an emergent operation should accordingly be performed.

## Data Availability

Data sharing is not applicable to this article.

## Abbreviations

CT: Computed tomography
IMA: Inferior mesenteric artery

## References

[1] Yoell JH (1959). Surprises in hernial sacs; diagnosis of tumors by microscopic examination. Calif Med..

[2] Boormans JL, Hesp WL, Teune TM, Plaisier PW (2006). Carcinoma of the sigmoid presenting as a right inguinal hernia. Hernia..

[3] Kouraklis G, Kouskos E, Glinavou A, Raftopoulos J, Karatzas G (2003). Perforated carcinoma of the sigmoid colon in an incarcerated inguinal hernia: report of a case. Surg Today..

[4] MacFadyen BV, Mathis CR (1994). Inguinal herniorrhaphy: complications and recurrences. Semin Laparosc Surg..

[5] Nicholson CP, Donohue JH, Thompson GB, Lewis JE (1992). A study of metastatic cancer found during inguinal hernia repair. Cancer..

[6] Benfatto G, Catania G, Tenaglia L, Lo Menzo E, Centoze D, Jiryis A (2006). Abscess and cecum carcinoma in inguinal hernia: case report. G Chir..

[7] Javors BR, Bryk D (1981). Colonic obstruction within inguinal hernia. J Can Assoc Radiol..

[8] Pappas D, Romeu J, Dave PB, Subietas A (1987). Colonic carcinoma in an inguinal hernia sac: case report and review of the literature. Mt Sinai J Med..

[9] Dewire DM, Bergstein JM (1992). Carcinoma of the sigmoid colon: an unusual cause of Fournier's gangrene. J Urol..

[10] Sakorafas GH, Peros G (2008). Obstructing sigmoid cancer in a patient with a large, tender, non-reducible inguinal hernia: the obvious diagnosis is not always the correct one. Eur J Cancer Care..

[11] Slater R, Amatya U, Shorthouse AJ (2008). Colonic carcinoma presenting as strangulated inguinal hernia: report of two cases and review of the literature. Tech Coloproctol..

[12] Ruiz-Tovar J, Ripalda E, Beni R, Nistal J, Monroy C, Carda P (2009). Carcinoma of the sigmoid colon in an incarcerated inguinal hernia. Can J Surg..

[13] Ko KH, Yu CY, Kao CC, Tsai SH, Huang GS, Chang WC (2010). Perforated sigmoid colon cancer within an irreducible inguinal hernia: a case report. Korean J Radiol..

[14] Tan A, Taylor G, Ahmed T (2013). Perforated sigmoid colon carcinoma in an irreducible inguinoscrotal hernia. Ann R Coll Surg Engl..

[15] Edmunds LH, Williams GM, Welch CE (1960). External fistulas arising from the gastro-intestinal tract. Ann Surg..

[16] Pernazza G, Monsellato I, Alfano G, Bacone B, Felicioni F, Ferrari D (2011). Laparoscopic treatment of a carcinoma of the cecum incarcerated in a right groin hernia: report of a case. Surg Today..

[17] Avidan B, Bardan E, Lang A, Fidder HH, Chowers Y, Bar-Meir S (2004). Colorectal cancer screening in patients presenting with an inguinal hernia: is it necessary?. Gastrointest Endosc..

[18] Avidan B, Sonnenberg A, Bhatia H, Aranha G, Schnell TG, Sontag SJ (2002). Inguinal hernia is not a sign of colon cancer: results of a prospective screening trial. Aliment Pharmacol Ther..

